# A multicenter, randomized controlled comparison of three renutrition strategies for the management of moderate acute malnutrition among children aged from 6 to 24 months (the MALINEA project)

**DOI:** 10.1186/s13063-018-3027-3

**Published:** 2018-12-04

**Authors:** Muriel Vray, Boris G. Hedible, Pierrick Adam, Laura Tondeur, Alexandre Manirazika, Rindra Randremanana, Halima Mainassara, André Briend, Cecile Artaud, Cassandre von Platen, Mathias Altmann, Ronan Jambou

**Affiliations:** 1 Unité d’Epidémiologie des Maladies Infectieuses, Institut Pasteur Dakar, Dakar, Senegal; 2 Unité des Epidémies et des Maladies Emergentes, Institut Pasteur, 25 Rue du Dr. Roux, 75015 Paris, France; 3 Unité d’Epidémiologie Institut Pasteur de Bangui, Bangui, Central African Republic; 4 Unité d’Epidémiologie, Institut Pasteur de Madagascar, BP1274, 101 Antananarivo, Madagascar; 5 Unité d’Epidémiologie CERMES, Niamey,, Niger; 6 Department of Nutrition, Exercise and Sports, Faculty of Science, University of Copenhagen, Rolighedsvej 30, DK-1958 Frederiksberg, Denmark; 7 Tampere Centre for Child Health Research, University of Tampere, Lääkärinkatu 1, 33014 Tampere, Finland; 8 Centre de recherche Transactionnel, Institut Pasteur, 28 Rue du Dr. Roux, 75015 Paris, France; 9 Action Contre la Faim, 14/16 Boulevard Douaumont – CS 80060, PARIS CEDEX 17, 75854 Paris, France; 10 Department of Parasites and Vector Insects, Institut Pasteur, 28 Rue du Dr. Roux, 75015 Paris, France

**Keywords:** Moderate acute malnutrition, Prebiotic, Azithromycin, Microbiota, Metagenomic

## Abstract

**Background:**

The aim of this open-label, randomized controlled trial conducted in four African countries (Madagascar, Niger, Central African Republic, and Senegal) is to compare three strategies of renutrition for moderate acute malnutrition (MAM) in children based on modulation of the gut microbiota with enriched flours alone, enriched flours with prebiotics or enriched flours coupled with antibiotic treatment.

**Methods:**

To be included, children aged between 6 months and 2 years are preselected based on mid-upper-arm circumference (MUAC) and are included based on a weight-for-height Z-score (WHZ) between − 3 and − 2 standard deviations (SD). As per current protocols, children receive renutrition treatment for 12 weeks and are assessed weekly to determine improvement. The primary endpoint is recovery, defined by a WHZ ≥ − 1.5 SD after 12 weeks of treatment. Data collected include clinical and socioeconomic characteristics, side effects, compliance and tolerance to interventions. Metagenomic analysis of gut microbiota is conducted at inclusion, 3 months, and 6 months. The cognitive development of children is evaluated in Senegal using only the Developmental Milestones Checklist II (DMC II) questionnaire at inclusion and at 3, 6, and 9 months. The data will be correlated with renutrition efficacy and metagenomic data.

**Discussion:**

This study will provide new insights for the treatment of MAM, as well as original data on the modulation of gut microbiota during the renutrition process to support (or not) the microbiota hypothesis of malnutrition.

**Trial registration:**

ClinicalTrials.gov, ID: NCT03474276 Last update 28 May 2018.

**Electronic supplementary material:**

The online version of this article (10.1186/s13063-018-3027-3) contains supplementary material, which is available to authorized users.

## Background

The main objective of this study is to improve the nutrition strategy used to treat moderate acute malnutrition (MAM). This is based on modulation of the gut microbiota with an adjunct product added to standard nutrition flours.

### Undernutrition in Africa

Child malnutrition in intertropical zones is a major public health problem. It favors the appearance of diarrhea but can also (1) disrupt the response to infection [1], (2) lead to an imbalance of the commensal flora and inflammation [2], and (3) modulate the response to vaccines [3]. These changes may also alter the cognitive development of children. Due to its life-threatening impact, programs provide care mainly for severe acute malnutrition (SAM), which is frequent in disturbed political contexts such as the Central African Republic (CAR) or during crop failure related to drought, like in Sahel. For example, in Niger, *Action Contre la Faim* Network (ACF) had to deal with 400,000 cases of SAM in 2013. However, aside from the emergency situation, SAM can develop in a context of chronic malnutrition (CM), such as in Madagascar, where 47.4% of children aged under 5 years are stunted [4]. MAM often happens during the first years of life when food is introduced in complement to breast milk. It can be managed at home and not at the hospital. However, if the management of the MAM changes over the last 30 years, the composition of flours has remained virtually unchanged. Indeed the time to recover is still long, and the percentage of success can be low [5], with high default rates or nonresponse to treatment, low coverage, and high associated costs [6–8].

### Malnutrition and microbiota

Related to poverty and poor access to sanitation, infectious diseases are responsible for 25% of deaths worldwide, and diarrhea is the third deadliest infection, with approximately 525,000 per year for children aged under 5 years [9, 10]. Diarrhea can modulate gut microbiota [11, 12] but an effect of an imbalance in the gut microbiota, regardless of diarrhea, on long-term child growth can also be important. This gut microbiota accounts for approximately 10^12^ microorganisms in humans, including bacteria, archae, yeast, viruses, and parasites. Gut microbiota can establish early in a child’s life (may be even during pregnancy and delivery) and it can modulate diseases [13]. It changes greatly with the introduction of food [14]. Its composition also varies between industrialized and low/middle-income countries (LMICs) with an increased abundance of *Prevotella* species in developing countries compared to an increased abundance of *Bacteroides* in developed countries. [15–17]. In LMICs, urease-positive strains are enriched, indicating adaptations to the low-protein food that is consumed [17].

Recent studies have shown a link between SAM and the colonic microbiota composition [18–24]. During the first years of life, children with SAM have a significant delay in the maturation of the microbiota [23]. Changes in microbiota composition lead to “dysbiosis,” marked by the enhancement of gut permeability and inflammation [25, 26]. Indeed, chronic inflammation induced by permanent stimulation due to food heavily contaminated by nonpathogenic or pathogenic bacteria could associated with villous atrophy, anorexia and malnutrition. These changes in microbiota could be a decrease in diversity or a shift in its composition as described in acute gastrointestinal infection [27, 28], inflammatory bowel disease [29, 30], coeliac disease [31–35], colon and liver cancer [36, 37], obesity [38], and diabetes [39].

The microbiota of non-stunted children looks enriched in probiotic species *Bifidobacterium longum* and *Lactobacillus mucosae*, whereas that of stunted children was enriched in inflammogenic taxa, including those in the genus *Desulfovibrio* and the order *Campylobacterales* [40]. Along the same line of reasoning, the metabolic impact of parasites such as *Isospora belli*, *Cyclospora*, *Cryptosporidium*, and *Microsporidia* in malnourished persons seems largely underresearched, but *Giardia* was recently associated with malnutrition [41].

During malnutrition, the disturbance of immunity may alter the response to vaccines, especially those administered orally (poliovirus, rotavirus, etc.) [2, 3]. This reduced efficacy was confirmed during severe malnutrition, but is still being debated regarding moderate and chronic malnutrition. The impact of microbiota on brain function is also a major focus of research [42–46].

### How to modulate the microbiota: antibiotics versus prebiotics

In malnourished children, clinical signs of infection can be attenuated. Indeed, the WHO recommends treatment of severely malnourished children with antibiotics [47]. Compared to standard flours, an increase in weight gain was described for children treated with flours + antibiotics [48]. In Malawi, a short-term effect of antibiotics on the nutritional recovery rate and mortality rate was confirmed for children with SAM [49, 50]. In Malawi, no effect of rifamixin on intestinal permeability was observed [49], whereas in Niger (Epicentre study), amoxicillin did not show any short-term benefit on the recovery rate of SAM compared with placebo [51]. However, hospital transfer rates decreased significantly. In Kenya, cotrimoxazole also failed to improve the clinical status of children with SAM [52]. The antibiotics can destroy gut microflora, but the effect on intestinal barrier integrity is still being debated [53].

Antibiotics can also restore equilibrium between the bacterial population induced by pathogenic bacteria. This has been described in the case of cholera, as a vibrio infection, and it results in a decrease in *Bacteroidetes*, *Firmicutes*, and *Actinobacteria* and an increase in harmful *Proteobacteria*. This disequilibrium was approached with treatment with macrolides; the abundance of *Escherichia coli*, *Enterococcus*, and *Veillonella* increased, while that of *Bifidobacteria*, *Bacteroides*, and *Ruminococcus* decreased [54].

However, the use of antibiotics for SAM children, even though recommended by the WHO, remains debated [55–59] both for its efficacy and for the possible increase in drug resistance [60]. To date, no trials have been performed on antibiotic use among MAM children. *Firmicutes* and other common gut microorganisms can contribute to fight by themselves against pathogens by local production of bacteriocins [61].

In the MALINEA (*Malnutrition et Infection de l’Enfance en Afrique*) protocol, macrolides were chosen to treat MAM based on (1) a narrower antibacterial spectrum of activity than other widely used antibiotics, (2) a rare use in dispensaries, especially as first-line treatment, which will prevent the emergence of resistant strains during mass administration, and (3) a favorable side-effect profile [62]. The main side effect of macrolides is gastrointestinal disturbance, which is less prevalent with azithromycin than with clarithromycin. For azithromycin, a long half-life and activity allows a 3-day treatment with a single daily dose (20 mg/kg/day). Anti-inflammatory and immunomodulatory activities have also been reported. A benefit was also reported on neonatal growth when given to the mother [63]. In line, the recent MORDOR trial [64] showed the benefit of azithromycin alone on the mortality and clinical status of children.

Microbiota can also be modulated through several very different compounds called probiotics [65], prebiotics, and milk oligosaccharides [66]. Microbiota transplantation can also be used but is still experimental [67].

For probiotics (a specific strain of bacteria inoculated orally to the patient), the choice of species and, even more, the choice of strain to use, are very important and remain pitfalls for clinical trials [68]. The benefit of combination of probiotics + prebiotics can also be questioned, as in the PRONUT study [69].

On the other hand, prebiotics are mostly nondigestible carbohydrates, which serve as substrates, and they support proliferation of certain bacteria, such as bifidobacteria and lactobacilli. Early in life, they are provided by maternal milk through breast-feeding at the same time as commensal bacteria [70].

Unlike antibiotics, prebiotics do not modify the existing strains of bacteria but impact the proportions of the different groups of bacteria in the intestine (gut microbiota). The best-known prebiotics are (1) fructans (fructose polymers including inulin), which are present in many plants and extracted from chicory tubers [71], (2) fructo-oligosaccharides (FOS), obtained by hydrolysis of inulin or by biosynthesis from sucrose, and (3) fructose/galactose-containing oligosaccharides (galacto-oligosaccharides, GOS), which are natural products of human breastmilk. In children, most studies have used combinations of inulin and GOS [72]. Depending on the microbiota, they reach more or less distal parts of the colon before being metabolized by the resident bacteria [73, 74]. Prebiotics have been associated in several studies with profound changes in the microbial ecology of the gut and in a shift towards “healthier” communities [75]. Many studies have been conducted in animals and in human adults [76], and recent studies support a beneficial effect for HIV-infected children. In studies performed so far using the prebiotic inulin, the proportion of *Lactobacillus* spp., *Bifidobacterium* spp. [77, 78], and *Faecalibacterium prausnitzii* [75, 79, 80] was shown to be increased during treatments, while the growth of *Enterobacteriaceae* and *Clostridium* spp. seemed to be restricted [78, 80, 81]. Prebiotics seemed to modulate lymphoid tissue associated with the intestine, resulting in improvement of inflammatory bowel symptoms in children or post-vaccine responses against measles [82–85]. Inulin consumption may improve the absorption of minerals in the colon, such as calcium, magnesium, zinc, and iron [86–88].

### Cognitive impairment

Malnutrition is frequently associated with cognitive delay, compromising child development and education [89, 90]. An effect of the modulation of microbiota in the development of cognitive function in children can be suspected [91, 92]. Deworming by itself can affect the cognitive skills of children [93] but this could be due to an indirect effect on iron and anemia. The opportunity for modulating the intestinal microbiota, diarrhea or cognitive development with pre- or probiotics is also under study [94–97].

### Malnutrition is a major health problem in the four countries involved in the project

The choice of the four countries in which to conduct this research is based on epidemiological data on moderate malnutrition, on existing facilities supported by ACF in the field and on the ability to perform high-level biological analysis in local laboratories. The countries selected in this project are particularly vulnerable to malnutrition and diarrhea. Programs are supported either by local facilities or through nongovernmental organizations (NGOs), especially ACF.

In Madagascar [4], acute malnutrition affects 8.6% of children aged under 5 years, including 1.4% with severe forms. At weaning, the majority of children benefit from the introduction of food supplements but have a poorly diversified diet. Growth retardation begins in utero (13% of children weigh less than 2.5 kg at birth) and increases at 12 months of age. Micronutrient deficiency is also a major public health problem in Madagascar that could increase the risk of morbidity and mortality [4].

In Niger, global acute malnutrition (GAM) has remained above the alert threshold (10%) in recent years among children aged 6–59 months. For children aged 6 to 23 months, malnutrition regularly exceeds the emergency threshold of 15%, as in 2005, 2010 (22%), and 2011 (20.2%). During the last 5 years, chronic malnutrition (CM) or growth retardation affected one of every two children in the country [97]. The overall underweight (weight- for-age) prevalence ranged between 32 and 40% over the past 5 years [98–101].

In 2014, in Senegal, the prevalence of GAM at the national level was 8.8%. The prevalence in some departments, such as Ranérou, Podor, and Kanel, exceeds the critical threshold of 15%. Sixteen departments of 45 have exceeded the threshold of 10% GAM. Chronic malnutrition affects 15.5% of children aged 0–59 months [102].

In the CAR, in 2012, at a national level, the GAM prevalence rate was 8%, and the SAM rate was 1.9%. The CM rate was 38.7% [103, 104]. In addition to the progressive reopening of nutrition centers, mobile clinics are organized to screen children among the displaced in Bangui (639,000 displaced people; i.e., 12% of the total population) [105]. The country is in crisis, and only NGO interventions cover these emergencies [106].

## Methods and design

### The aim

The aim of this project is to define an easy-to-use modified strategy of renutrition to use in primary health centers to treat MAM before it degenerates into SAM. The main problem to tackle is that, in a peri-urban context, alimentation of small children is difficult to manage accurately, especially when food (or financial) resources are limited.

### The objectives

The general objective of this project is to improve nutrition for children with moderate malnutrition using strategies to modulate intestinal microbiota. The objective of this three-arm, open-label, randomized multicountry study is to evaluate the effectiveness of two improved strategies of management of MAM in children aged between 6 months and 2 years in comparison with a reference standard of care. Recovery is defined by a (weight-for-height Z-score) WHZ ≥ − 1.5 standard deviations (SD) after 3 months of treatment.

The secondary objectives are (1) to compare, for each group, all the measurements before and 3 months after intervention (weight, length, mid-upper-arm circumference (MUAC)); (2) to compare, between the three groups, the occurrence of clinical outcomes (intercurrent illness, hospitalization and transfer to the next level of care), the compliance to protocol, and tolerance (reported adverse and side effects, duration and intensity); and (3) to analyze the gut microbiota of the children during the renutrition process. In Senegal, psychomotor development will be assessed at inclusion and 3, 6, and 9 months after intervention using the DMC II (Developmental Milestones Checklist). DMC II scores will be compared (1) for the same child before and after intervention, (2) at inclusion between matched healthy children and malnourished ones from the same district, and (3) between arms at 3, 6, and 9 months.

### The study design (protocol version 2_ 28 February 2018)

According to centralized randomization, the children will receive one of the following:

- Arm 1 (control): malted milk *Prise en Charge de la* MAM (PEC-MAM) flour + antiparasitic (albendazole)
- Arm 2 (antibiotic): malted milk PEC-MAM flour + antiparasitic (albendazole) + azithromycin for 3 days
- Arm 3 (prebiotic): malted milk PEC-MAM flour + antiparasitic (albendazole) + inulin/FOS mixed with flour

A control group of well-nourished children without diarrhea aged 18 to 24 months will also be included to match the malnourished ones but without follow-up visits. The objective is to investigate microbiota of well-nourished children living in the same place and at the same time as the case group, to take into account local and seasonal variation of the microbiota. As only one stool sample will be obtained from non-stunted children, children under 18 months will not be included to discard the fluctuating composition of the microbiota at such young ages.

### Participants and enrollment

In Niger, the study will be conducted in the department of Mayahi, in the municipalities of Tchake and Issawanne. In Tchake, the total population is 28,338 inhabitants, with 5668 children aged less than 5 years, and in Issawanne, the total population is of 45,152 inhabitants, with 9030 children aged under 5 years.

In Senegal, recruitment will be conducted in the district of Guédiawaye, near Dakar. The district is a low-income area of 355,525 inhabitants, with an average rate of MAM of 6%. The primary health care center will be *Hamo V*, where an average of 500 MAM cases are followed each semester. A large team of community relay agents, led by nurses from the primary health care center, is in charge of the screening of MAM in the community.

In Madagascar, the study will take place in poor neighborhoods of Antananarivo. Two different structures run by local NGOs (CRENAM Andohotapenaka) and Centre de Santé Mitia are dealing with malnourished children.

In the CAR, the study will take place in Bangui in two private centers that support MAM: the St. Jacques and the St. Joseph’s Health Centres. These centers are supported by the World Food Program, which provides nutritional supplements.

Prescreening of malnourished children aged between 6 and 24 months will be conducted in the country by community health workers using arm circumference (115 ≤ MUAC < 130 mm). To be definitively included, children should have a WHZ between − 3 and − 2 SD, and should be followed for at least 3 months. Written consent from the parents or legal guardians will be obtained. Children not satisfying these criteria will not be included, nor will those (1) with complications requiring intensive clinical care (severe vomiting, hypothermia < 35 °C or fever > 39 °C, pneumonia, and severe anemia), (2) with extensive infections, (3) who are weak, apathetic or unconscious, and (4) or presenting fitting/convulsions, severe dehydration or any condition that requires an infusion or tube feeding. Children currently receiving one of the following treatments will also be excluded from entering the study: antacids, cetirizine, digoxin, ergotamine, zidovudine. Moreover, those with hypersensitivity to macrolides, flour or the prebiotics used during the study will also be excluded. HIV testing is not planned as its prevalence is very low in three countries out of four. For CAR systematic testing of young children for HIV was considered as a stigmatization of the mother by the National Ethic Committee which will induce refusal of the mothers to participate.

### Recruitment, randomization, and follow-up (Fig. 1 and Additional file 1)

The enrollment will take place at the primary health center after weight and height assessment, signature of written consent from the parent or legal guardian, and verification of inclusion/exclusion criteria.

**Fig. 1:**
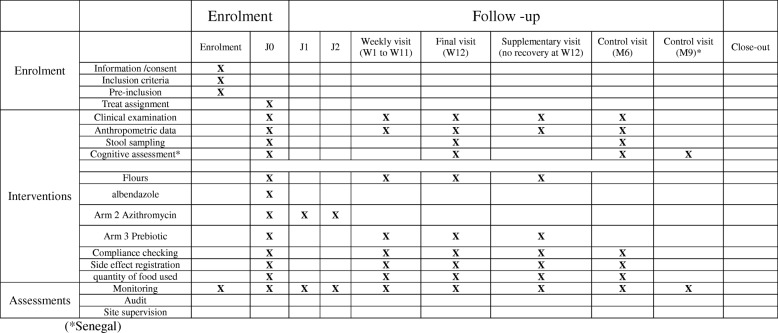
Timetable of the follow up of the children

Randomization will be stratified by country, health center, and age (6–11 months versus 12–24 months). Randomization will be achieved in a 1:1 ratio using random permutation blocks. A clinical examination will be done, and stool samples will be collected. Systematic treatment with albendazole will be given to all children in the three arms aged older than 12 months after collection of the stools. For children treated with azithromycin, the first treatment will be given after stool collection at the dispensary. Doses for the following 2 days will be given at the health center as well.

After enrollment, in Senegal, children with MAM will be evaluated for cognitive development and ability, with a DMC II (Developmental Milestones Checklist) questionnaire applied by a trained social worker.

Children will be followed up weekly for 12 weeks and at 6 months (M6) at the health center. At each visit, (1) flours will be given to the caretaker for the whole week, (2) weight, height, and arm circumference will be assessed, and (3) a clinical examination will be carried out, and data on safety and child’s food consumption will be investigated. In the case of a missing visit or insufficient weight gain, a visit at home will be organized by health workers to collect supplementary information.

At inclusion, 3 months (M3) and 6 months (M6), stool samples will be collected. After 6 months, the follow-up will be stopped except for in children involved in the psychomotor development evaluation protocol, who will be followed until age 9 months (M9). However, in the case of nonrecovery at M3, children will still receive follow-up care according to the national recommendations but will be considered “treatment failure.”

### Interventions

#### Supplementary feeding

Default of quality of flours obtained from the local market in African countries is a very common pitfall that is poorly addressed by NGOs. For the trial, flours will be produced by an industrial partner *(*PROTEIN KISSEE LA *Zone industrielle, Vridi, Port-Bouet, 18 BP 2335 Abidjan*). It is a well-established brand on the West African market providing ready-to-eat fortified complementary foods. The composition of the flours, described in Table 1, will be adapted to the international malted milk PEC-MAM standard [107].

**Table 1:** Composition of the flours

	Without prebiotics	With prebiotics	
Composition (% of dry weight)			
wheat	62.4%	60.9%	
soya	20.8%	20.3%	
sugar	8%	8%	
dry milk	7%	7%	
iodinated salt	0.2%	0.2%	
vitamins and salts	1.3%	1.3%	
flavor	0.4%	0.4%	
Prebiotic	0%	2%	
Total	100%	100%	
Overall composition of the flour and additional compounds (for both with and without prebiotic): composition per 100 g of flour			
calories (kcal)	420	ascorbic acid	60
protein total (g)	15	thiamin	1
lipids (g)	9	riboflavin	1
carbohydrates	66	nicotinamide (mg)	12
fibers	2	folic acid (mg)	0.3
sodium (mg)	200	pantothenic acid (mg)	4
calcium mg)	266	biotin (μg)	0.016
phosphorus (mg)	200	vitamin E (IU)	10
potassium (mg)	610	vitamin B12 (μg)	1.8
iron (mg)	23.2	vitamin A (IU)	2668
copper (mg)	0.18	vitamin D (IU)	400
zinc (mg)	16.6	vitamin B6 (mg)	1
manganese (mg)	2.4		

#### Antibiotics

A vial of powder for oral suspension of azithromycin (600 mg) will be restored at 200 mg/5 mL and then given with a syringe for oral administration three times once a day over a maximum of 4 days (in case of 1 day missing) (20 mg/kg/day daily for 3 days). The same batch of antibiotics will be used in the four countries.

#### Prebiotics

The recommended doses with positive effects on the intestinal flora are between 2.5 to 10 g/day [108, 109]. The main side effects are observed with doses more than 40 g/day, as the substances can cause bloating and induce intestinal cramps. Abdominal pain has been reported with doses of 10 g/day. For this study, we will evaluate the effectiveness of a combination of prebiotic inulin / FOS (Synergy1 6 g/day if age ≥ 12 months and 4 g/day if < 12 months), as it is simple to be obtained (manufactured by BENEO, Tienen, Belgium) and is active at different levels of the digestive tract.

### Data collection

#### Clinical data

At inclusion, a standardized questionnaire will be conducted with the parents or legal guardians to collect general characteristics, socioeconomic and clinical data.

At each visit, clinical assessment will be recorded until recovery and then at M6, data will be registered concerning undercurrent illness, signs of infections (diarrhea, vomiting, fever, cough), need to transfer to hospital, and safety and adherence to treatments (defined as completion of all 7 days of the study regimen).

#### Anthropometric data

The children will be monitored weekly for 3 months (or until recovery if treatment fails) at the primary health center. One additional visit is planned at 6 months. At each visit, weight, length, and MUAC will be measured. Balances will be from SECA UNICEF (± 100 g). Length will be measured on children placed in an elongated position using a Fathom Infant / Child ShorrBoard for infants aged less than 2 years (130 cm with graduations of 0.1 cm). MUAC will be measured with a UNICEF MUAC tape.

#### Laboratory processes

Stool samples will be collected during the day and stored at 4 °C until transportation to the laboratory. At laboratory, stools will be separated in aliquots and stored at − 80 °C until analysis. Stool examination will be based on metagenomic approaches. Whole deoxyribonucleic acid (DNA) will be extracted from stools using a procedure adapted for stools including bead fractioning and a QIAGEN stool extraction kit. Fragments of the 16S gene will be polymerase chain reaction (PCR) amplified using V3/V4-adapted primers. Sequencing of the PCR products will be carried out on a Miseq Illumina machine. Metagenomic analysis will be conducted using the SHAMANN package. In parallel, direct detection of pathogens (*Salmonella*, STEC, *Campylobacter*, *Vibrio cholerae*, *V parahaemolyticus*, *V vulnificus*, *Cryptosporidium* sp., *Entamoeba histolytica*, *Giardia lamblia*, Microsporides sp., Adenovirus, Astrovirus, Norovirus, Rotavirus) will be done using specific quantitative-PCR (Q-PCR) and ELISA protocols in the *Institut Pasteur de Madagascar, Institut Pasteur de Dakar*, and *Institut Pasteur de Bangui.*

#### Cognitive and motor development

During this study, a Developmental Milestones Checklist II (DMC II) questionnaire will be administered to participating children to evaluate their cognitive and motor abilities. This questionnaire is adapted to children aged between 1 and 59 months and has been developed specifically to be used in developing countries and particularly in sub-Saharan Africa.

In Senegal, the DMC II will be applied during enrollment at inclusion and 3, 6, and 9 months, after enrollment. Eighty children with MAM and 80 healthy non-stunted children will be included in this specific phase. This questionnaire has already been used in several countries (Kenya, Bukina Faso, Mali). It can be applied by trained but unspecialized field workers and is fast to document (20–30 min). Adaptation of the questionnaire to the local context will be done during a preliminary study conducted at the Children’s Hospital of Dakar.

### Evaluation criteria

The primary endpoint is recovery, defined by WHZ > − 1.5 SD at two successive examinations without hospitalization or undercurrent discharge to SAM or lost to follow-up. The main endpoint will be at 3 months. This is a stronger criteria of recovery than usual (WHZ > − 2), which avoids misclassification of the children at the end of the intervention.

Secondary outcomes include anthropometric parameters (weight, length, and MUAC), time for recovery, and safety of the strategy, compliance to treatment and psychomotor development evolution (for Senegalese children).

The main biological secondary endpoint is to analyze the variations of microbiota and the presence of specific pathogens during the renutrition process. The major question is to understand if microbiota differ between well- and malnourished children and if renutrition retardation is related to a specific type of microbiota. Microbiota will be expressed as Operational Taxonomic Units (OTUs), and the results of Q-PCR as positive/negative.

### Statistical considerations

Full details on data management and the database can be available on reasonable request to the corresponding author. Databases and confidentiality purposes were already approved by either the National Ethics Committees, the Pasteur Institute IRB or the French Office for Safety in Data Meaning (CNIL).

#### Sample size

Based on previous studies showing a recovery rate of between 50 and 88%, we can assume a likelihood of nutritional recovery in the control arm of approximately 60% after 12 weeks. To show a benefit of at least 15% with antibiotics or prebiotics, with a type I error of 0.017 (Bonferroni correction for multiple comparisons), a power of 90% and a two-sided test, 840 children will be enrolled (280 per arm), with 210 in each country (with an allowance to loss of 10%). After enrollment, in Senegal, 80 children with MAM will be evaluated and followed for their cognitive development and ability.

#### Statistical analysis

The primary endpoint will be analyzed using both the intention-to-treat population (children lost to follow-up or referred at hospital considered as failures) and the per- protocol population (including only children without protocol deviation). A third analysis will be conducted using the last value available (endpoint analysis). A chi-squared test will be used to compare the primary endpoint (recovery at 3 months) between the three groups.

To take into account the time to recovery, a survival analysis will be performed using a Cox model with adjustment for center and age. Comparisons will be also performed for the other anthropometric criteria (weight, height, MUAC, weight gain) as well as for a DMC II test using a mixed linear model for repeated measurements.

#### Bioinformatic analysis

For the stool samples, bioinformatics analysis will be conducted to identify the species present in each sample. As many species can exist in the same sample, samples will be clustered in “profiles” of composition using principal component analysis. In the first step, these profiles will be considered classes of a parameter called “microbiota.” However, some specific species could impact the renutrition process more deeply. A second round of analysis will be conducted using the species themselves, taking into account the comparison of groups of children with rapid versus slow recovery. At the time of inclusion (day 0), analysis will focus on (1) comparison of microbiota between countries, (2) association of some socioeconomic parameters with some specific profile of microbiota (all countries together), and (3) differences in microbiota between children with or without malnutrition (for children aged over 18 months). During the time course of the renutrition process, comparison of microbiota will be done (1) between the arms of treatment, (2) between children with and without recovery, and (3) between children with slow and fast recovery.

## Discussion

Child malnutrition is a major public health problem in intertropical zones, but most of the resources and studies focus on SAM due to its life-threatening impact. However, SAM develops most often with chronic malnutrition that can lead to moderate acute malnutrition during shortening of family resources or during child infections, before worsening into SAM. MAM thus has a heavy impact on national health structures, as the number of children involved is usually higher than that for SAM, and acting effectively on MAM could prevent having to treat more SAM. The delay in seeking treatment is also often long, causing the situation to worsen and complicating treatment. Compliance to treatment is also a challenge. Facing these problems, almost no change in the strategy of treatment has been validated for the last 30 years. Decreasing the time of treatment and improving the efficacy could be major achievements.

MAM occurrence is almost exclusively restricted to the first 24 months of life, closely relating to the time when foods complementary to breast milk are introduced. However, recent studies on gut physiology have drawn attention to the role of dis-equilibrated gut microbiota both in malnutrition and obesity. We decided to use two strategies (prebiotics and antibiotics) to modulate the microbiota while providing renutrition with flours.

A short-term effect of antibiotics on nutritional recovery rate was observed in children treated for severe malnutrition. The effect on MAM could be similar. On the other hand, prebiotics serve as substrates and induce proliferation of bifidobacteria and lactobacilli, changing the proportions of different groups of bacteria in the gut. Other strategies could be envisioned, such as the use of anti-oxidants, specific micronutrients, etc.

Overall, we currently have only a partial understanding of interactions between nutrients, gut microorganisms and the immune system. Thus, we have chosen a pragmatic approach for the MALINEA study with two strategies targeting the gut microbiota in a broad manner. They are safe and practical to use at the community level in LMICs. Considering the large number of children concerned, criteria such as low prices, ease of use, and potential availability of local production, are very important for the choice of a strategy.

This three-armed randomized intervention will be evaluated by effects both on the children’s anthropometric parameters and on their microbiota, providing first insights into the underlying mechanisms linking MAM, nutritional recovery and microbiota composition. This study received approval from the National Ethic Committees of the four host countries as well as from the Internal Review Board of the Pasteur Institute in Paris.

### Trial status

ClinicalTrials.gov, ID: NCT03474276 Last update on 28 May 2018.

## Additional file

Additional file 1: Standard Protocol Items: Recommendations for Interventional Trials (SPIRIT) 2013 Checklist: recommended items to address in a clinical trial protocol and related documents*. (DOC 122 kb)

## Abbreviations

ACF: *Action Contre la Faim*
CAR: Central African Republic
CM: Chronic malnutrition
CRENAM: *Centre de RENutrion re-Adaptation des Malnutris*
DMC II: Developmental Milestones Checklist II
FOS: Fructo-oligosaccharides
GAM: Global acute malnutrition
GOS: Galacto-oligosaccharides
MALINEA: *Malnutrition et Infection de l’Enfance en Afrique*
MAM: Moderate acute malnutrition
MUAC: Mid-upper-arm circumference
NGO: Nongovernmental organization
OTU: Operational Taxonomic Unit
PEC-MAM: *Prise en Charge de la* MAM
Q-PCR: Quantitative polymerase chain reaction
SAM: Severe acute malnutrition
SD: Standard deviation
WHZ: Weight-for-height Z-score
