# Correction to: A multicenter, randomized controlled comparison of three renutrition strategies for the management of moderate acute malnutrition among children aged from 6 to 24 months (the MALINEA project)

**DOI:** 10.1186/s13063-019-3339-y

**Published:** 2019-04-11

**Authors:** Muriel Vray, Boris G. Hedible, Pierrick Adam, Laura Tondeur, Alexandre Manirazika, Rindra Randremanana, Halima Mainassara, André Briend, Cecile Artaud, Cassandre von Platen, Mathias Altmann, Ronan Jambou

**Affiliations:** 1 Unité d’Epidémiologie des Maladies Infectieuses, Institut Pasteur Dakar, Dakar, Senegal; 2 Unité des Epidémies et des Maladies Emergentes, Institut Pasteur, 25 Rue du Dr. Roux, 75015 Paris, France; 3 Unité d’Epidémiologie Institut Pasteur de Bangui, Bangui, Central African Republic; 4 Unité d’Epidémiologie, Institut Pasteur de Madagascar, BP1274, 101 Antananarivo, Madagascar; 5 Unité d’Epidémiologie CERMES, Niamey, Niger; 6 Department of Nutrition, Exercise and Sports, Faculty of Science, University of Copenhagen, Rolighedsvej 30, DK-1958 Frederiksberg, Denmark; 7 Tampere Centre for Child Health Research, University of Tampere, Lääkärinkatu 1, 33014 Tampere, Finland; 8 Centre de recherche Transactionnel, Institut Pasteur, 28 Rue du Dr. Roux, 75015 Paris, France; 9 Action Contre la Faim, 14/16 Boulevard Douaumont – CS 80060, PARIS CEDEX 17, 75854 Paris, France; 10 Department of Parasites and Vector Insects, Institut Pasteur, 28 Rue du Dr. Roux, 75015 Paris, France

**Correction to: Trials (2018) 19:666**

**https://doi.org/10.1186/s13063-018-3027-3**

After publication of the original article [1], the authors have notified us of an additional acknowledgement they wish to bring for their paper.

The phrase to be added to the Acknowledgement section is:

- We also thank Pascale Vonasch from Institut Pasteur Paris and Antonio Varga from Action Against Hunger, Spain for their expert contribution in the text and in the proof-reading of the last version.
